# More or less likely to offend? Young adults with a history of identified developmental language disorders

**DOI:** 10.1111/1460-6984.12339

**Published:** 2017-11-21

**Authors:** Maxine Winstanley, Roger T. Webb, Gina Conti‐Ramsden

**Affiliations:** ^1^ School of Health Sciences The University of Manchester Manchester UK; ^2^ Manchester Academic Health Sciences Centre (MAHSC) Manchester UK

**Keywords:** offending, young adults, developmental language disorders, outcomes, police contact, substance use

## Abstract

**Background:**

There is now substantial literature demonstrating that a disproportionate number of young people who come into contact with youth justice services evidence unidentified language difficulties. These young people, therefore, have received little or no professional input in this area. Conversely, there is a dearth of research pertaining to criminality outcomes among those individuals with identified developmental language disorders (DLD) who have received such interventions.

**Aims:**

To examine police‐initiated contact and substance use outcomes of young adults with a history of identified DLD versus age‐matched peers (AMP). Additionally, self‐reported rule breaking behaviours and aggression are considered. We hypothesize that early identification/intervention reduces engagement with risky behaviour such as substance and alcohol use as well as offending‐related behaviours.

**Methods & Procedures:**

Adversarial police‐initiated contacts were examined in 84 young adults with a history of DLD and 88 AMP. Rule‐breaking and aggression were evaluated using the Achenbach Adult Self‐Report for ages 18–59 years.

**Outcomes & Results:**

Adults with a history of DLD who received targeted intervention during their school years reported less contact with their local police service compared with AMPs at age 24. Comparable proportions of both groups reported current alcohol consumption, but group differences were found relating to alcohol use. No group differences in rule‐breaking behaviours were found, but the DLD group was found to have a statistically significant higher raw score on the aggressive behaviour scale.

**Conclusions & Implications:**

There is a need for early identification of children with DLD. Early intervention aimed at ameliorating such difficulties could possibly have distal outcomes in relation to offending.

**What this paper adds**

**What is already known on the subject**

A high number of young offenders display language abilities across multiple domains that are well below that of the typical population. Furthermore, these language difficulties have previously gone unrecognized.

**What this paper adds to existing knowledge**

This study reports evidence that young adults with a history of DLD, who have received early targeted intervention aimed at ameliorating such difficulties, report less contact with their local police than their AMPs. We argue that targeted early intervention could possibly mitigate against the negative effects of language difficulties, diverting these individuals away from involvement in crime.

**What are the potential or actual clinical implications of this work?**

The findings of this study underline the importance of timely assessment, early identification and subsequent targeted intervention for children with DLD.

## Introduction

A substantial body of literature exists demonstrating that a disproportionate number of young people who come into contact with youth justice services (YJS) have developmental language disorders (DLD) (Bryan *et al*. 2007, 2015, Lount *et al*. 2017, Snow and Powell 2005, 2008). Although direct comparisons are complicated by methodological issues, even in the most conservative estimates (Snow and Powell 2011) rates are markedly raised. Children and young people can experience problems with language for many reasons: they can be associated with other neurobiological disorders such as autism, hearing impairment or learning difficulties, or they can be the child's main area of difficulty. Historically different diagnostic terminology has been used to describe the children in this latter category whose language difficulties are not accounted for by physical, cognitive and/or neurological causes (Durkin and Conti‐Ramsden 2010), including language impairment (LI), DLD and specific language impairment (SLI). Longitudinal studies in this area, e.g. the Manchester Language Study (MLS), have also reflected in their publications, the historical changes in terminology used with this population (Conti‐Ramsden and Botting 1999). In line with current recommendations, following a Delphi consensus study focusing on characteristics, diagnosis and terminology in this area (Bishop *et al*., 2016a; Bishop *et al*., 2016b) this paper will use the term ‘DLD’ throughout and retain it when considering background literature. Although these difficulties are first discernible in childhood, longitudinal studies have highlighted that they persist into adolescence and adulthood (Beitchman *et al*. 2001, Clegg *et al*. 2005). The current prevalence of DLD is approximately 7% (Tomblin *et al*. 1997). However, not all young people are identified by professionals as having language needs and therefore many do not receive intervention or support for their difficulties.

### Language abilities and young offenders

Despite sampling variation and distinct measures and cut‐off points employed to delineate DLD, one striking similarity is that the language difficulties of young people who come into contact with YJS have previously gone undetected (Bryan *et al*. 2007). This finding is consistent across Western countries (Bryan *et al*. 2015, Sanger *et al*. 2003, Snow and Powell 2005). In the UK, Bryan *et al*. (2007) reported that scores on standardized tests obtained by 58 young people chosen at random in a UK young offenders’ institution indicated language deficits. The mean age of the group was 17 years, yet the mean score achieved on the British Picture Vocabulary Scale (BPVS‐II; Dunn *et al*. 1997), a test of receptive vocabulary, was 11.5 years. No participant gained a score consistent with their chronological age. The authors reported that between 46% and 67% of participants scored within the ‘poor’ or ‘very poor’ range (that is, in the lower 9% of the general population) in the four subtests used (listening vocabulary, listening grammar, speaking vocabulary and speaking grammar on the Test of Adolescent and Adult Language—TOAL; Hammill *et al*. 1994). This suggests a high number of the participants in the study would meet criterion for DLD. When the authors considered the degree to which the group was performing below average on the TOAL, it was found that the figures increased to between 66% and 90% of participants.

Bryan *et al*. (2015) administered subscales of the Clinical Evaluation of Language Fundamentals, 4th Edn (CELF‐4; Semel *et al*. 2006) to a sample of 118 young offenders. They found receptive language more severely affected than expressive language, with 42% scoring 1.5 standard deviations (SD) below the mean on a measure of receptive language and 21% recording similar low scores on measures of expressive language. Just over one‐third scored 1.5 SD below the mean when receptive vocabulary abilities were examined using the BPVS‐II (Dunn *et al*. 1997). Receptive language difficulties are often hidden (Durkin and Conti‐Ramsden 2010) and much more difficult to recognize in everyday interactions as individuals can use context to aid comprehension. Moreover, in a youth justice setting, these receptive difficulties may manifest as rudeness or uncooperativeness. Such behaviours may further disadvantage the young person (Snow *et al*. 2016).

When comparing the oral language skills and social skills of young offenders placed on community orders against a control sample from local schools, Snow and Powell (2008) reported that the offenders performed significantly worse than the control group on all language and social skill measures, but not on measures of nonverbal IQ. They identified that over 50% of the group presented with clinically significant DLD, which could not be accounted for by nonverbal IQ. Furthermore, the authors ruled out socio‐economic status (SES) as a mediating factor by utilizing a similar SES comparison group. The recognition that one in two young people displayed DLD was replicated when the authors conducted a cross‐sectional study of 100 incarcerated young people (Snow and Powell 2011).

The primary sentencing disposal for young people in England and Wales who plead guilty is a Referral Order (RO) (Edwards 2011). This order requires the young person, and their guardian, to attend a referral order panel (ROP) consisting of youth offending team (YOT) staff, community panel members and the victim (should they chose to attend). It is, therefore, paramount that young offenders form a coherent narrative of an incident, with the desired amount of sincerity, so that others can make sense of the surrounding events. Provision of a clear well‐constructed narrative is, therefore, a skill needed to aid successful passage through the youth justice system as it allows a young person's story to be heard (Snow *et al*. 2012). In a sample of young male offenders completing community based orders compared with demographically matched non‐offending youths, Snow and Powell (2005) measured narrative discourse by asking the youths to ‘tell the story of what happened’ in their own words after being shown a six‐frame cartoon stimulus known as The Flowerpot Incident. The authors considered not only the quantity and the quality of the output but also structural adequacy in terms of story grammar. The picture stimulus remained in view during the assessment thereby accounting for memory as a confounding factor. The young offenders were found to respond to each picture in turn rather than formulate important extrapolations between characters’ internal feelings and their following actions. The use of such a piecemeal strategy is likely to result in relational incoherence as ideas are not connected, thereby creating difficulties for the listener to determine any conceptual relationships. Thus, the young offenders’ performance on the narrative tasks yielded results suggestive of considerable difficulties explaining thoughts, interpreting motives and identifying elements serving as precursors to the resolution of the story.

The studies detailed thus far have comprised exclusively of male participants. Although fewer authors have considered the language abilities of female offenders, those that have report a higher prevalence of DLD than in the general population (Sanger *et al*. 2001). In the United States, Sanger and colleagues assessed 67 incarcerated females aged 13–17 years. They reported that 19% of the sample scored 1.3 SDs below the mean on the CELF‐3 (Semel *et al*. 1995), therefore meeting the clinical criteria for a diagnosis of DLD (Sanger *et al*. 2001). In a recent cross‐sectional study, considering the language, emotion recognition and mental health of 100 incarcerated young people, the authors reported that DLD was present in 27% of the females in the study (Snow *et al*. 2016).

When interviewing incarcerated females with a history of DLD, participants revealed experiencing difficulties understanding curricular vocabulary and following directions at school (Sanger *et al*. 2003). In a similar vein, Hopkins *et al*. (2016) conducted qualitative semi‐structured interviews with 31 young people on court orders. Unfortunately, this study did not include any language measures so it was unknown if the participants had DLD. The authors, however, reported that the young offenders divulged difficulties listening at school, and some described an inability to comprehend large segments of information (Hopkins *et al*. 2016). It is likely such difficulties would have compromised engagement and motivation in the classroom (Sanger *et al*. 2000). Left unchecked this can lead to disengagement with education and alliance with similar disconnected peers (Gifford‐Smith *et al*. 2005). Such findings demonstrate the impact on behaviour that language limitations can exert. Following completion of standardized language tests, researchers have reported that a significant proportion of young people referred to child and adolescent mental health services, have previously undetected DLD (Cohen *et al*. 1993). The authors concluded that the psychopathology was possibly secondary to the DLD.

Taken together the available research strongly suggests that young offenders, as a group, are likely to have significant language problems that have not been previously diagnosed. They perform poorly on standardized language measures of vocabulary, grammar and narrative. It appears that a proportion of young offenders have unidentified language difficulties and have thus received no professional support for their language difficulties.

### Early intervention and potential socio‐emotional distal beneficial impacts

Head Start (HS) is a public pre‐school programme exclusively aimed at disadvantaged families in the United States. Its original aims were to reduce the educational gap between children reared in poverty and their more affluent peers (Garces *et al*. 2002). The theory underpinning the programme lies in the compensatory hypothesis, which posits that the most marginalized, disadvantaged children will benefit the most from targeted early intervention (Sameroff and Chandler 1975).

The short‐term benefits for children participating in HS include improved standardized test results (Karoly *et al*. 1998), equal to an increase of 0.15–0.35 SDs (Ludwig and Philips 2008), in areas such as receptive vocabulary and phonemic awareness (Barnett and Hustedt 2005). The Head Start Impact Study (HSIS) (Puma *et al*. 2010) corroborated such findings reporting significant impacts on the cognitive and socio‐emotional development of participants. The largest impacts were found in language and literacy outcomes as measured by Peabody Picture Vocabulary Test (PPVT; Dunn and Dunn 1997) and the Woodcock–Johnson III letter–word identification test (Woodcock *et al*. 2001). The HSIS, however, concluded that programme's effect attenuated by age 7. It appeared that the results of HS investment were short lived.

However, recent longitudinal studies (Carneiro and Ginja 2014, McKelvey *et al*. 2015) reported ‘unexpected’ favourable longer‐term, distal outcomes in those who had participated in HS in that they demonstrated fewer parent–child dysfunctional interactions (McKelvey *et al*. 2015), less depression, less obesity, fewer behavioural problems (Currie and Neidell 2007) and less offending (Carneiro and Ginja 2014). The developmental theoretical perspective is rooted in the notion that early childhood intervention can prevent later costly problems in adolescence and adulthood that appear unrelated to the content of the childhood intervention (Moffitt *et al*. 2011, Sprague and Walker 2000).

A body of evidence is now accumulating pertaining to the long‐term impact of the HS initiative. Garces *et al*. (2002) utilized a within‐family methodology comparing children who did benefit from the scheme with their siblings who did not. They found that statistically significantly more HS children completed high school, were more likely to attend college and less likely to be involved in offending (Garces *et al*. 2002). Although the authors made use of within‐family methodology, there remains the possibility that within‐child factors may contribute to the findings.

Utilizing data from the children of the national longitudinal survey of youth (CNLSY), Carneiro and Ginja (2014) investigated the impact of HS intervention across adolescence and young adulthood at three time points: ages 12–13, 16–17 and 20–21 years. At the earliest of these age ranges multiple variables were considered across three different domains: cognition, behaviour and health. For the other two age ranges behaviour was the main focus. The comparison group consisted of children also from low income families, but who either attended different preschool care or who were cared for in the home. Consistent with the childhood findings of HS any differences in cognitive measures had attenuated by time 1. However, significant health and behaviour benefits were associated with the HS group such as a reduction in obesity, the prevalence of chronic conditions and behaviour problems as measured by the Behaviour Problems Index (BPI; Centre for Human Resource Research 2000). At age 20–21 years there were significantly fewer arrests and convictions among males in the HS group. These findings have been replicated in the United States with other cohorts such as the Chicago Child–Parent Program (Reynolds *et al*. 2001).

These distal effects have also been noted in other initiatives aimed at high‐risk families. The Perry Preschool study began in 1962 and randomly assigned 123 African American children, born in poverty, to one of two groups (Schweinhart *et al*. 1993). The groups were matched on gender, SES and baseline IQ. One group received intensive, high quality education five mornings a week and the other group received no pre‐school education. Data were collected at multiple waves, up to, and, including age 40. The age 27 data revealed a significantly higher level of schooling, employment rates, monthly earnings, and significantly fewer arrests for those young people who had received the early education intervention (Schweinhart *et al*. 1993).

A childhood intervention that targeted academic, social skills and self‐control entitled Fast Track, involving a total of 891 children deemed as aggressive and high risk at the start of elementary school, also provides interesting results at follow‐up in early adulthood (Sorensen and Dodge 2016). Children were divided into two groups: 445 of them received the intervention and 446 made up a control group. Findings at age 20 indicated that fast track participants were 39% and 34% less likely to have been arrested as a juvenile and as an adult respectively (Conduct Problems Prevention Research Group 2010). Analysis aimed at identifying the components of intervention responsible for this effect illustrated that improvements in social skills and self‐control had the biggest impact. Sorensen and Dodge (2016) reported standard coefficient of 0.19 for interpersonal skills and a standard coefficient of 0.23 for intrapersonal skills. Mediation analysis of the Perry Preschool intervention found similar results (Heckman *et al*. 2013). In the UK there have been few studies on distal, potentially beneficial outcomes of early interventions. Goodman and Sianesi (2005) found positive educational and employment outcomes in adulthood for individuals who had received early pre‐school education (but also see Apps *et al*. 2013).

Finally, it should to be noted that potential benefits may be offset by the presence of other risk factors for offending. Young people known to YJS have often experienced lives marred by socio‐economic disadvantage (Stephenson 2007). The literature exploring low SES and DLD suggests that growing up in poverty may be a risk factor for language difficulties (Locke *et al*. 2002) by virtue of type and frequency of language exposure (Hart and Risley 1995). Indeed, in areas of lower SES the prevalence of DLD has been reported to increase to 31% (Enderby and Pickstone 2005). This socio‐economic disadvantage continues into adolescence (Spencer *et al*. 2012). SES typically depends on a combination of variables and researchers have used a variety of these to delineate the construct in samples including individual‐level factors such as education, income and occupation, as well as ecological area‐level measures assigned via residential postcode.

A number of factors have been recognized as contributors to offending behaviour. Strong associations have been found between offending and exposure to violence (Darker *et al*. 2008, Widom and Maxfield 2001), substance use (Biederman *et al*. 2008) and alcohol misuse (Richardson and Budd 2003). Furthermore, early substance use increases the risk for misuse and later dependence (Van Ryzin and Dishion 2014). Tobacco use, in contrast, lacks this strong association with long‐term offending or aggression. Associations between tobacco use and delinquent behaviour have been limited to adolescence (Ellickson *et al*. 2001) and have attenuated over time (Tucker *et al*. 2008). Any associations with crime that have been reported have arisen from unadjusted analysis which failed to take into account potential confounders that could account for the relationships found (Mathers *et al*. 2006).

### The present study

There is a dearth of longitudinal studies that have reported on offending in young people with identified DLD. This is particularly surprising given what we know of the high prevalence of unidentified language difficulties in the young offending population. As reviewed above, few studies conducted in the UK have examined potential beneficial long‐term outcomes for individuals who have received specialist intervention in childhood. To our knowledge, this is the first investigation to examine offending and behaviours associated with offending, such as alcohol and substance use, in a group of young adults with a history of DLD who attended language units and received intensive early intervention in childhood (referred to henceforth as young adults with a history of identified DLD). Our main prediction was that early language intervention programmes, such as those provided by language units, have long‐term improved distal outcomes. These potential benefits could include less frequent engagement with risky behaviours such as substance and alcohol use as well as reductions in offending‐related behaviours such as contact with the police, rule‐breaking and aggression.

Specifically, this paper addresses two research questions:

- Is substance use lower in young adults with a history of identified DLD compared with age‐matched peers (AMPs)?
- Are contact with the police, rule‐breaking behaviours and aggression scores lower in young adults with a history of identified DLD compared with their AMPs?

This investigation utilized data from the MLS, a study of individuals who attended language units in England in early childhood. The study we have reported on in this paper focused on young adults aged 24 years.

## Method

### Ethics

The study reported here received ethical approval from the University of Manchester. All participants provided their informed written consent.

### Participants

#### Young adults with a history of identified DLD

Participants were originally part of the longitudinal MLS, which consisted of an initial cohort of 242 children (77% male, 23% female) (Conti‐Ramsden *et al*. 1997). These children represented a random sample of 50% of all 7‐year‐olds attending 118 language units across England for at least half of the school week. Language units are specialized classes equipped to meet the needs of children who have been recognized as having DLD, that is, language is their main area of difficulty and their language difficulty is not associated with other neurobiological disorders such as autism. To be placed in a language unit, children generally have to fulfil a number of criteria. Most units in England require children to have, or be undergoing assessment for, a statement of special educational needs or, from 2014, an education, health and care plan. This document details their difficulties and the professional input they require including intensive speech and language intervention. Language units have a high staff to pupil ratio and provide input from both speech and language therapists and specialized teachers. This level of support is reserved for children who would be unable to cope or would struggle to access the curriculum in a mainstream setting, even with the support of a teaching assistant.

Individuals were contacted again at ages 8 (*n* = 232), 11 (*n* = 200), 14 (*n* = 113), 16 (*n* = 139) and 24 (*n* = 84). Funding constraints contributed to attrition at subsequent follow‐up stages of the study. The current sample, 35% of the original cohort, consisted of 56 (67%) males and 28 (33%) females, ranging in age between 23.4 years and 25.9 years (mean = 24.4; SD = 0.7 years). To examine potential attrition bias, we compared the receptive language, expressive language, nonverbal IQ and gender distribution of individuals with a history of DLD who continued to participate at 24 years and those who did not. There were no significant differences in receptive language (*t*(240) = −1.13, *p* = .261), expressive language (*t*(229) = −0.45, *p* = .654), and nonverbal IQ (*t*(231) = −0.60, *p* = .547) standard scores at age 7 between those who participated at age 24 and those who did not. At age 24 years, the gender distribution in the DLD group (67% male; 33% female) was not significantly different from the gender distribution of the comparison group (56% male; 44% female, see below), *χ* ^2^(1, *N* = 172) = 2.18, *p* = .140.

#### Aged‐matched peers (AMPs)

The comparison group consisted of 88 aged‐matched peers, 49 (56% were males) and 39 (44%) were females, ranging in age between 22.3 years and 26.0 years (mean = 24.1; SD = 0.9 years). The comparison group had no history of speech and language therapy provision or provision for special educational needs (as ascertained by teachers’ reports). Sixty‐six of these young adults were recruited at age 16 years and 22 young adults were recruited for the final wave of the MLS. The age 16 participants were recruited from the same schools as the participants with a history of DLD as well as additional targeted mainstream schools. Specific demographic areas were selected to recruit comparison peers from broadly similar social backgrounds to the participants with a history of DLD. Care was taken so that the 22 young adults that participated in this study matched the original sample in terms of age and socioeconomic status as measured by personal income. All participants had remained in school until the end of compulsory education (in the UK, at 16 years on average). The DLD and the AMP groups did not differ on household income at age 16 years (*χ* ^2^(10, *N* = 145) = 9.32, *p* = .501). In previous research with the MLS it has been established that the participating families came from a broad range of social‐economic backgrounds with percentages of both DLD and AMP participants in each income band category distributed similarly across the SES range (as measured by maternal education and household income; Wadman *et al*. 2008). Participants also did not differ on personal income at age 24 years (*χ* ^2^(5, *N* = 131) = 7.38, *p* = .194). In addition, comparisons were carried out between the DLD and AMP groups on additional key social factors at age 16 including maternal education, other languages spoken at home and whether parent was a homeowner. We found no significant differences across those with and without DLD (table 1).

**Table 1:** Comparison of DLD and AMP groups on social factors at age 16 years

	DLD, *n* (%)	AMP, *n* (%)	Test statistic
Mother achieved at least one GCSE qualification?	62 (47%)	57 (48%)	*χ* ^2^(1, *N* = 251) = 0.07^NS^
Mother achieved at least an A‐Level qualification?	56 (42%)	54 (46%)	*χ* ^2^(1, *N* = 251) = 0.34^NS^
Mother achieved at least a university qualification?	19 (14%)	20 (17%)	*χ* ^2^(1, *N* = 251) = 0.34^NS^
Other languages spoken at home?	10 (7%)	6 (5%)	*χ* ^2^(1, *N* = 254) = 0.55^NS^
Parent homeowner?	99 (75%)	95 (82%)	*χ* ^2^(1, *N* = 248) = 1.72^NS^

Note: NS, not significant. Values represent the number of participants answering yes, with per cent in parentheses.

### Materials and measures

#### Substance use

Alcohol consumption and drug use were included in this domain. Participants were asked a series of questions concerning their use of alcohol. Those who stated they consumed alcohol were then asked to give an estimate of the volume and frequency of their consumption. Participants were also asked about whom they drank alcohol with. Four choices were offered; alone, with family, with friends or with strangers. These questions were also used to establish any drug use among the sample and if so to assess the extent of this drug use (Appendix A lists the questions used in the study).

#### Contact with the police

Participants were asked a series of questions pertaining to adversarial police‐initiated contacts. In line with the Offending Crime and Justice Survey (OCJS), which develops models of police‐initiated contacts (Ariza 2014), key variables were explored. Respondents were asked to declare if (1) they had ever been in trouble with the police (hereafter TWP), (2) they had ever been told off or asked to move on by the police (hereafter ATM), (3) stopped and searched by the police (hereafter SAS), (4) stopped but not searched (hereafter SNS), (5) if they had ever received a warning or caution, and finally 6) if they had ever been arrested. Questions required a yes/no response.

#### Rule‐breaking and aggression

Rule‐breaking and aggression were evaluated using the Achenbach Adult Self‐Report for ages 18–59 (ASR; Achenbach and Rescorla 2003). This measure consists of normed sets of scales each containing multiple subscales. Participants were asked to rate their behaviour over the past 6 months on a three‐point scale (0 = not true, 1 = somewhat or sometimes true, 2 = very true or often true). The rule‐breaking behaviour scale consisted of 15 items which were summed to create a raw score. Questions included (1) I break rules at work or elsewhere and (2) I lie or cheat. The aggressive behaviour scale also consisted of 15 items which were summed to create a raw score, questions included (1) I get in many fights and (2) I threaten to hurt people. Higher scores correspond to a greater number of symptoms. Previous research has shown the ASR to be a valid measure of externalizing difficulties in this age group (Rescorla and Achenbach 2004).

#### Smoking

Unlike substance use and alcohol misuse, tobacco use is not strongly associated with long‐term offending or aggression. Thus, smoking can be considered as a control outcome measure in the design of this study. That is, childhood intervention would not be expected to have beneficial impact on tobacco use in young adulthood. Those participants that confirmed they smoked tobacco were then asked a series of questions. These questions established the onset age of smoking behaviour, frequency per week and amount smoked per day.

#### Assessment of language and nonverbal skills

To assess language ability, the Clinical Evaluation of Language Fundamentals (CELF‐4^uk^; Semel *et al*. 2006) was utilized. The CELF‐4 is a standardized assessment and is normed up to age 21 years 11 months. Three subtests of the CELF‐4 were utilized, these consisted of word classes receptive (WCR) which requires the participant to listen to a list of four words and decide which two are related. Formulating sentences (FS) requires the participant to formulate a sentence, including a given word, based on a picture shown. This measures the ability to articulate in a coherent logical order illustrating both vocabulary use and sentence structure. A further receptive measure was used, understanding spoken paragraphs (USP). Where the WCR relies on the ability to comprehend associations among words, and is concerned with the structural aspect of language, USP focuses on an individual's ability to process, comprehend and formulate a response to verbally presented information. This entails answering questions that test not only factual information but also inferential understanding. For the age range 17;0–21;11 years, the reliability of the WCR subtest was .88, FS subtest was .82, and, of the USP subtest was .75. Clinical validation studies of the CELF‐4 reported in the manual indicate that the test is sensitive to DLD in children, adolescents and young adults. None of the participants in this study reached ceiling in the CELF‐4 tasks.

The Wechsler Abbreviated Scale of Intelligence (WASI; Wechsler 1999) Performance subscale was administered as a measure of nonverbal IQ and standard scores were calculated. This test has norms for individuals aged 6 to 89 years. The reliability of the Performance IQ scale for the age range 20–24 years is .94. Validity studies of the WASI reported in the manual provide evidence that the test is a valid quick screening measure of intellectual functioning.

### Data handling and statistical analysis

The data were analysed using SPSS Version 20. Comparisons between groups were based on the two‐sample *t*‐test for continuously scaled data or non‐parametric Mann–Whitney *U*‐test where data were not normally distributed. Pearson's Chi‐square was utilized for dichotomous and ordered categorical variables. Although we posit directional hypotheses for long‐term impact of early intervention, we are mindful of the dearth of studies specifically looking at individuals with a history of DLD. We thus have adopted the more rigorous (non‐directional) two‐tailed approach as we are not able to rule out that the results could in fact go in the other direction than the one posited. The threshold for statistical significance was set at *p *< .05. For subgroups where risk of police contact was indicated by chi‐squared tests to be statistically different between the DLD and AMP groups, we estimated risk ratios from log‐binomial regression models, with 95% CIs obtained using a normal approximation and the standard error of the log (risk ratio), as described in Altman (1991).

## Results

### Psycholinguistic profiles of the participant groups

Comparisons of mean standard scores pertaining to language and nonverbal IQ for DLD versus AMP, including SDs, are presented in table 2. All scores for the AMPs were within the expected range. The mean language scores for the young adults with a history of identified DLD fell below 1 SD below the mean (< 0.85). Mean nonverbal scores were within the expected range and close to the population average. Participants with a history of identified DLD, however, had significantly lower nonverbal IQ scores than their peers (Leonard 2014).

**Table 2:** Psycholinguistic profiles of the DLD and AMP groups

	Group					
	DLD (*N* = 84)	AMP (*N* = 88)	*t*	d.f.	Mean difference [95% CI]	Cohen's *d*
WCR	83.51 (18.60)	106.22 (8.94)	10.17***	168	22.71 [18.30–27.11]	1.56
FS	81.57 (18.93)	105.64 (12.07)	9.89***	167	24.07 [19.26–28.88]	1.52
USP	70.90 (14.17)	86.74 (13.03)	7.57***	167	15.84 [11.71–19.97]	1.17
Nonverbal IQ	98.80 (15.80)	111.93 (10.28)	6.43***	167	13.14 [9.10 to −17.17]	1.08

Notes: ^***^ *p *< .001. All scores are standard scores means and standard deviations in parentheses.

DLD, developmental language disorder; AMP, aged‐matched peers.

### Substance use

A high percentage (84.3%) of the young adults in the study reported that they currently consumed alcohol. No significant between‐group differences were found (*χ* ^2^(1, *N* = 172) = 2.56 *p* = .11), with comparable proportions of the DLD and AMP groups reporting current alcohol consumption (80% (67/84) and 87% (78/88) respectively. Between‐group differences were found, however, in relation to alcohol use with AMP reporting significantly more days drunk, consuming more alcohol units per session and an earlier onset age of drinking behaviour. These results are presented in table 3.

**Table 3:** Characteristics of alcohol use by DLD and AMP group

	DLD (*N* = 67)			AMP (*N* = 78)			
Measures	Mean	SD	Range	Mean	SD	Range	*p*‐value Mann–Whitney *U*‐test
Age started drinking alcohol	16.9	2.16	8–23	15.6	1.78	11–21	<.001
Days drunk in the last 6 months	5.4	13.5	0–96	12.3	13.1	0–60	<.001
Total alcohol units consumed per session	9.32	6.95	1–39	13.2	8.33	1–47	.001

Note: DLD, developmental language disorder; AMP, aged‐matched peers.

**Figure 1:**
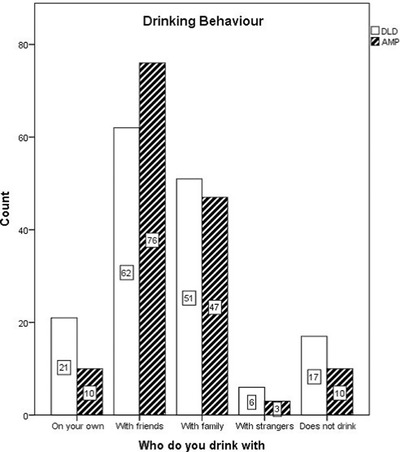
Drinking behaviour by DLD and AMP group.

Participants were asked about their socializing habits when drinking and with whom they chose to drink alcohol. This is summarized in figure 1. The totals exceed participant numbers as some fell into more than one category. It was found that over two thirds (68%) of the participants who reported drinking alone came from the DLD group and this difference was significant (*χ* ^2^(1, *N* = 145) = 7.36 *p* = .007). No further significant between group differences were found.

A total of 15 (9%) participants reported that they participated in drug use, for reasons other than medical use. Among those that reported drug use, 13% (2/15) were from the DLD group and 87% (13/15) were from the AMP group, this difference was statistically significant, Fisher's Exact Test *p* = .005. The low level of drug users in the DLD group prevented further analyses but, interestingly, onset age for drug use was higher in the AMP group (mean = 17.6) compared with the DLD group (mean = 16.5).

Recall that smoking was examined due to its lack of robust association with offending behaviour. There was a statistical trend for a higher percentage of smokers to be AMP, 27% (24/88) rather than young people with identified DLD, 16% (13/84), *χ* ^2^(1,172) = 3.54 *p* = .06. Both groups of participants began smoking at approximately the same age (mean = 16.19, SD = 2.52 for DLD and mean = 17.08, SD = 2.75 for AMPs), *t*(44) = 1.30, *p* = .198 and smoked similar number of cigarettes per day (median = 9, SD = 17.3 for DLD; and median = 7, SD = 6.81 for AMPs), *t*(35) = 1.18, *p* = .244.

### Contact with the police, rule breaking and aggressive behaviours

The percentage of participants reporting TWP significantly differed by group status (DLD versus AMP), *χ* ^2^(1, *N* = 172) = 6.75, *p* = .009. The risk ratio indicated that participants in the AMP group were almost two‐and‐a‐half times (risk ratio = 2.44, 95% CI = 1.1–4.9) more likely to have been in TWP. Among the 32 participants that reported being in TWP, 68.8% were male. A higher proportion of males reported TWP and this was true for both groups; 67% males (6/9) for the DLD group and 70% males for the AMP group (16/23).

The percentage of participants that declared that they had been ATM by the police also significantly differed by group status (DLD versus AMP), *χ* ^2^(1, *N* = 169) = 15.14, *p *< .001. The AMPs were just over three times more likely to have been ATM on by the police (risk ratio = 3.13, CI = 1.7–5.9). Although the trend was for AMPs to report a higher frequency of SAS, SNS, warnings/cautions and arrests, these failed to reach significance. Descriptive statistics and chi‐square analysis relating to contact with the police are summarized in table 4.

**Table 4:** Frequency of police contacts by DLD and AMP group

	DLD			AMP					
Measure	*N*	Frequency	%	*N*	Frequency	%	Chi‐square	*p‐*value	Risk ratio [95% CI]
TWP	84	9	10.7	88	23	26.1	6.75	.009	2.44 [1.20–4.97]
ATM	81	10	12.3	88	34	38.6	15.1	<.001	3.13 [1.65–5.92]
SAS	81	10	12.3	88	16	18.2	1.10	.293	
SNS	81	13	16.0	88	28	22.7	1.20	.274	
Warnings/cautions	81	6	7.4	88	13	14.8	2.30	.130	
Arrested	81	4	4.9	88	9	10.2	1.66	.197	

Note: TWP, trouble with the police; ATM, asked to move on or told off; SAS, stopped and searched; SNS, stopped and not searched; DLD, developmental language disorder; AMP, aged‐matched peers.

The mean score for the aggressive behaviour scale was lower (indicative of fewer problems) for the AMP group than for the DLD group. Results indicated that the DLD group had a statistically significant higher raw score (*U* = 2380, *p* = .037). However, the percentage of individuals that fell within the borderline or clinical range for aggression was not statistically significant across groups, (13% for DLD versus 7% for AMP), *χ* ^2^(1, *N* = 167) = 1.51, *p* = .219. There were also no group differences in rule breaking behaviours between the two groups. Details are presented in table 5.

**Table 5:** Aggressive and rule breaking behaviour by DLD and AMP group

	DLD (*N* = 80)			AMP (*N* = 80)			
Measures	Mean	SD	Range	Mean	SD	Range	*p*‐value Mann–Whitney *U*‐test
Aggressive Behaviour	6.18	5.58	0–23	4.32	4.13	0–17	.037
Rule breaking behaviour	2.45	2.59	0–13	2.53	2.95	0–12	.784

Note: DLD, developmental language disorder; AMP, aged‐matched peers.

## Discussion

This is the first published study of the relationship between identified DLD and offending in a UK context, to the best of our knowledge. Our findings revealed that young adults with a history of identified DLD do not appear to have an elevated risk of contact with local police officers or a higher arrest rate. In contrast to the literature pertaining to young people with unidentified DLD, and in line with our prediction, individuals with a history of identified DLD in this investigation reported less contact with the local police service compared with AMPs. This study is consistent with the United States literature, reviewed earlier, that has considered programmes implemented to address the needs of the most vulnerable. In line with this literature, individuals who have previously received intervention for their DLD reported statistically significant less trouble with the police and less instances of being moved on by police officers. Although perhaps considered a less intrusive police‐initiated contact, it results in the young person becoming known and recognized by local police officers. In our TWP and ATM model, group effect was statistically significant. For the other police contact related variables the effect was in the expected direction: AMP reporting higher levels.

In terms of aggression, participants with a history of identified DLD evidenced significantly higher aggression scores than AMPs. It is now well established that young people with DLD are at increased risk of experiencing social, emotional and behavioural difficulties (Durkin and Conti‐Ramsden 2010, Lindsay and Dockrell 2012, St Clair *et al*. 2011). A recent meta‐analysis reported that children with DLD were more than twice as likely to exhibit externalizing problems than their typically developing peers (Yew and O'Kearney 2013). Aggressive behaviour has been significantly associated with violent offending (Ang *et al*. 2011) and is a predictor of delinquency and violence (White *et al*. 1990). Snow and Powell (2011) reported an elevated rate of DLD in young offenders convicted of more serious offences. As part of the longitudinal Ottawa Language Study, Brownlie *et al*. (2004) examined whether 19‐year‐old young adults with a history of speech and language impairment evidenced higher levels of aggression and delinquency compared with their peers. Parent reported aggression symptoms were higher in the DLD group. Self‐reported arrests and convictions for males were also higher in the DLD group than in their peers. However, the history of childhood language intervention for individuals in this community based sample was not examined as a potential influencing factor on the findings on delinquency. In this study, no between group difference was found for clinical level symptoms of aggression. Results are indicative of elevated subclinical levels of aggression for those with a history of identified DLD. It is important to note, however, that this aggression did not manifest in rule‐breaking behaviours in young adulthood for these individuals. It could be hypothesized that higher levels of support received in the language units provided the children with enhanced strategies, equipping them to deal with, and therefore mitigate later problem behaviour.

Explanations that can account for this increase in aggression scores may include higher levels of frustration, confusion and inability to utilize linguistic skills as a facilitator to navigate and effectively solve disputes. Children with DLD display significantly more withdrawn behaviours at school (Fujiki *et al*. 2001). Interaction difficulties with peers are common for children and adolescents with DLD, resulting in reduced positive social interactions and feedback (Mok *et al*. 2014). In the context of typical language development, individuals engage in interaction and develop their social skills. Young people with DLD have often been excluded from these practice opportunities thus impacting on language acquisition (Durkin and Conti‐Ramsden 2010). Young people who are unable to create and negotiate a peer network due to compromised social interaction skills are more likely to associate with people already involved in crime (Quinton *et al*. 1993). Instances of peer rejection, even at moderate levels, continuing for 1–2 years, predict adolescent antisocial involvement (Laird *et al*. 2001). Furthermore, the time at which such behaviour commences can affect the longevity, with the later the onset the greater the ability to desist from antisocial behaviour (Piehler and Dishion 2007). It is possible that young people who cannot rely on language skills for positive socialization may turn to delinquent behaviour to gain social status when language demands exceed their abilities.

A significantly higher number of AMPs reported drug use. Only two members of the DLD group reported using drugs. Age of onset of this use was lower for the adults with a history of identified DLD, although this needs to be interpreted with caution due to the small number of drug users in this sample. No group difference was found relating to the number of participants that consumed alcohol. In a longitudinal community sample of youths with and without early childhood speech and language difficulties, Beitchman *et al*. (1999) also found no group differences in the rates of alcohol and drug use. The authors suggested that DLD provided a protective factor against antisocial behaviour that usually occurs in groups. Our study takes this suggestion further. We suggest that language difficulties that have been identified and supported by intervention may lead to less risky behaviour in relation to alcohol and drug use. Evidence for this suggestion was also found when we examined the frequency and intensity of consumption. On occasions when young adults chose to consume alcohol, participants in the AMP group reported consuming a significantly greater amount of alcoholic units and also reported spending significantly more days under the influence of drink in the preceding 6 months. Although significantly more members of the DLD group reported drinking alone there was no difference for drinking with friends. This would suggest that members of the DLD group participated in social drinking but they consumed less alcohol than the AMP. This is likely to result in less problematic behaviour fuelled by alcohol and less contact with the police. Nonetheless, it is important to acknowledge that these young people may have small social networks, and that this may be the underlying reason for reduced use. Further research in this area is certainly warranted.

The growing body of evidence detailing unrecognized DLD in the youth offending population suggests that the full consequences of coping with poor language skills during the developing years and the associations with offending behaviour may only be realized later in life (Brownlie *et al*. 2004). Scant research exists concentrating on crime related variables in adults with a history of DLD. The one other study exclusively focusing on offending and DLD utilized data from the Danish National Crime Register to compare prevalence, and type of offence, between cases of identified DLD and comparison peers from the general population (Mouridsen and Hauschild 2009). Individuals at a mean age of 37.5 years were compared on a range of crime outcome measures. Their findings indicated a 3.3% lower conviction rate for young adults with identified DLD. This reduction in convictions, however, was not statistically different than that observed in peers. Poor levels of language proficiency have been associated with literacy and numeracy difficulties (Snowling *et al*. 2001), low educational attainment (Conti‐Ramsden and Durkin 2012), social stress (Wadman *et al*. 2011) and social, emotional and behavioural difficulties (Durkin and Conti‐Ramsden 2010, St Clair *et al*. 2011). These highly interwoven issues lead Bryan *et al*. (2015) to suggest a ‘compounding risk model’, whereby poor language results in further risks. For those whose DLD has remained unrecognized it is likely that, without the necessary modifications to the curriculum, they will have faced significant challenges in the classroom keeping up with curricular vocabulary, listening and following classroom instructions (Sanger *et al*. 2000). Teachers may see the presentation of a young person's language difficulties as rudeness, egocentricity or a lack of cooperation (Snow and Powell 2011). All these difficulties are likely to affect motivation and engagement, potentially resulting in problem behaviours. Moreover, academic difficulties can lead to disengagement with the education system and alliance with other disconnected peers (Gifford‐Smith *et al*. 2005), which further perpetuates problems.

We found that for young adults whose language difficulties had been identified resulting in experiencing specialist intervention in language units, outcomes were more favourable. It is possible that such interventions are offering long‐term benefits with cascading effects on other life course domains not directly targeted by the intervention. Further evidence for this is that the outcomes were more favourable than for AMPs, young adults who had not developed with language difficulties. This allows for the consideration that the intervention procured benefits that over time have contributed to the prevention of later adverse crime outcomes. Therefore, the interventions aimed at ameliorating language difficulties may also not only offer children the opportunity to develop strategies to adjust socially and emotionally to the long‐term nature of their language difficulties, but also improve the young person's competence in other areas associated with offending such as emotional self‐regulation (Snow *et al*. 2016), above and beyond their typically developing peers. A young person's involvement in offending behaviour is usually due to multifactorial issues, which can include, but are not limited to social disadvantage, educational underachievement and mental health (Bryan *et al*. 2015, Snow *et al*. 2016). These issues could have contributed to the higher incidences of offending in the AMP group. The findings of this study demonstrate the importance of assessment, identification and appropriate intervention for children and young people with DLD. The language difficulties experienced by the young people currently in the criminal justice system have gone unrecognized and unsupported. A central reason for unmet need is the lack of appropriate and timely assessment (Harrington and Bailey 2005). This investigation provides novel evidence that specialist intervention in childhood has the potential to disrupt risk and reduce the cycle of adverse outcomes in relation to substance use and rule breaking.

A significant issue for policymakers is the cost–benefit ratio of initiatives such as specialist intervention for DLD. In other words, does the investment of financial resources yield sufficient results? Often only short‐term benefits are examined with little consideration given to the broader long‐term implications and societal savings. Intervention for a 16‐year‐old with DLD has been estimated to cost £200,000, however incarceration increases this by £100,000 (Hartshorne 2006). Any type of contact with the criminal justice system incurs substantial cost, with approximately £1000 million spent on ‘processing and dealing with young offenders’ (Barratt *et al*. 2006). Due to the time elapsed since these calculations this is likely to be an underestimation of current costs. Offending is costly to society and has an extensive impact on public resources, direct and indirect victims (such as family members), and the wider community (Marder 2013). Therefore, innovative approaches to divert young people away from criminality continue to be sought. Routine assessment and targeted intervention, especially for those at‐risk groups, is a strategy that warrants empirical investigation (Snow and Powell 2008).

### Limitations

As with all longitudinal research, there remains the possibility that unmeasured variables may have contributed to the between‐group differences observed. In addition to substance and alcohol use, the literature details many factors that have been recognized as important correlates of offending behaviour and a discussion of these is beyond the scope of this article. It is possible that factors including childhood maltreatment (Caspi *et al*. 2002), exposure to violence (Darker *et al*. 2008) and delayed psychosocial maturity (Baskin‐Sommers and Newman 2014) could have contributed to group differences. Research including other potential influencing factors is needed. These findings relied on self‐reported data. Although young adults may over report offending behaviour, due to a perception of higher social status, or underreport for fear of disapproval, previous research has found self‐report to be accurate (Thornbury and Krohn 2001). We also acknowledge that there are validated self‐report tools for obtaining data on alcohol consumption and drug use. However, given the multiple areas of functioning examined in this phase of the MLS, we were under time constraints which resulted in focusing on a small number of questions which are reported in this investigation. Future research using a number of measurement tools as well as multiple sources of data, for example police records, would provide further insight into the relationships among language difficulties, early identification and early interventions, and socio‐emotional outcomes in early adulthood. Additionally, future research would benefit from the inclusion of a comparison group that consists of young people with DLD who do not receive early intensive intervention aimed at ameliorating these difficulties.

## Conclusions

This is the first study to examine potential beneficial distal effects of childhood identification and intervention for DLD in relation to substance use, contact with the police and rule‐breaking in young adulthood. It is well established that a high number of young offenders display language abilities across multiple domains that are well below that of the typical population. These language difficulties have, for the most, gone previously unrecognized and earlier opportunities to identify and intervene have been lost. Regularly monitoring the language abilities of children who display problem behaviours or who underperform in school could be a target for prevention efforts. Speech and language therapy is deemed a vital service in settings that involve those with high rates of DLD (Law *et al*. 2013), moreover, there is evidence to suggest that speech and language intervention is effective within the YJS (Gregory and Bryan 2011). Provision embedded within the youth justice settings would allow for identification of DLD upon entry and a better awareness among practitioners of the nature and implications of such difficulties.

Findings from research such as ours have important implications for practice. They support the need for early identification and intervention for children with DLD. With respect to policy, our results speak to the notion that intervention efforts aimed at ameliorating language difficulties could possibly have distal outcomes in relation to offending that positively alter the trajectory of these young people.

## Appendix A Questions asked pertaining to alcohol and drug use

*Alcohol use*

1. Do you currently drink alcohol?
2. How old were you when you started drinking alcohol?
3. On average, how often do you have a drink containing alcohol?

Only a few times a year/About once a month/Once a fortnight/One or two days per week/Three or four days per week/Five or six days per week/Every day/Rather not say

4. In the past 6 months, on how many days were you drunk
5. Do you think the amount of alcohol you drink is?

About right/Too much/Not enough

6. When you drink alcohol, is it:

On your own/With friends/With family/With strangers

7. Which is your preference?

*Drug use*

1. Do you use drugs other than those required for medical reasons?
2. How old were you when you started taking drugs?
3. In the past 6 months, on how many days did you use drugs for non‐medical purposes?
4. Who do you take them with?

On your own/With friends/With family/With strangers

5. Which is your preference?
